# Two Decades (2003–2024) of Investigating *Sarcocystis* in Thrushes (*Turdus* spp.)

**DOI:** 10.3390/pathogens15070709

**Published:** 2026-07-07

**Authors:** Eglė Rudaitytė-Lukošienė, Liuda Kutkienė, Saulius Švažas, Dalius Butkauskas, Petras Prakas

**Affiliations:** State Scientific Research Institute Nature Research Centre, Akademijos Street 2, LT-08412 Vilnius, Lithuania; liudbir@gmail.com (L.K.); saulius.svazas@gamtc.lt (S.Š.); dalius.butkauskas@gamtc.lt (D.B.); prakaspetras@gmail.com (P.P.)

**Keywords:** *Sarcocystis*, *Turdus*, molecular identification, ITS1, *cox1*, phylogeny, host specificity

## Abstract

*Sarcocystis* spp. are apicomplexan parasites that form sarcocysts mainly in the muscles or central nervous system of intermediate hosts and sporocysts in the intestines of definitive hosts. Three species, *Sarcocystis falcatula*, *Sarcocystis calchasi* and *Sarcocystis halieti*, are potentially pathogenic to their intermediate hosts. Over the past two decades, we have examined 72 thrushes across four species (redwing (*Turdus iliacus*), common blackbird (*Turdus merula*), song thrush (*Turdus philomelos*), and fieldfare (*Turdus pilaris*)) for sarcocysts to better understand their role as intermediate hosts. Sarcocysts were detected in 28 individuals (38.9%). Most sarcocysts observed by light microscopy were of a single morphological type consistent with *Sarcocystis turdusi*. A molecular analysis of ITS1 sequences confirmed the presence of *S. turdusi* in the common blackbird, song thrush, and fieldfare, establishing the latter two bird species as new intermediate hosts. In contrast, *cox1* was not a sufficiently variable locus for species differentiation. Additionally, a single sarcocyst with a smooth cyst wall, distinct from *S. turdusi*, was detected in a common blackbird and identified as *S. halieti* based on ITS1 sequence analysis. This atypical host record represents an isolated finding in a long-term dataset. Further sampling is required to confirm its epidemiological significance.

## 1. Introduction

Members of the genus *Sarcocystis* (Apicomplexa: Sarcocystidae) are cyst-forming coccidian parasites that infect mammals, reptiles, and birds. These parasites are characterised by an obligatory two–host predator–prey life cycle [1]. The parasites undergo asexual multiplication in the intermediate host, resulting in the formation of sarcocysts, primarily in the striated muscle or central nervous system. Sexual stages (oocysts/sporocysts) develop in the small intestine of the definitive host. Some *Sarcocystis* species are pathogenic to their intermediate hosts. Pathogenicity mainly depends on the parasite species, location in the host, infection dose, and the host’s immune state. Birds may serve as intermediate or definitive hosts for numerous *Sarcocystis* species [2]. Current evidence suggest that *Sarcocystis falcatula*, *Sarcocystis calchasi* and *Sarcocystis halieti* are potentially pathogenic to their avian hosts. These three species are characterised by the formation of sarcocysts in multiple hosts across several bird orders; among them, the pathogenicity of *S. halieti* in hosts has not been thoroughly studied. To date, only granulomatous encephalitis associated with *S. halieti* has been established in the little owl (*Athene noctua*) [3].

*Sarcocystis* species are usually described in intermediate hosts. The main phenotypic diagnostic criterion for *Sarcocystis* species is the structure of the sarcocyst wall [1]. Sarcocysts are examined morphologically using light and electron microscopy, and morphologically similar sarcocysts from different species can be found in the same intermediate host species or even within a single animal. Thus, morphological data is combined with DNA sequence analysis for the description and differentiation of *Sarcocystis* species. For molecular analysis, DNA is usually extracted from individual sarcocysts isolated from host tissues, artificially digested or minced tissue, or unprocessed host tissues. DNA isolation from excised sarcocysts is a favourable approach as it allows morphological and molecular examination of the same sarcocyst; however, it requires a high level of researcher competence. Previous studies have revealed that internal transcribed spacer 1 (ITS1) is the best genetic locus for discriminating relatively recently evolved avian *Sarcocystis* species [4].

Thrushes (Passeriformes: Turdidae: *Turdus* spp.) host a diverse range of parasites, including ectoparasites, helminths, and protozoans, reflecting their broad ecological niches and wide geographic distribution [5,6,7]. Thrushes have been suggested as potential bioindicators of environmental contamination, particularly due to their ground-feeding behaviour, which may increase their exposure to pollutants such as microplastics [8,9].

The song thrush (*Turdus philomelos*) is a widespread and abundant species in Lithuania, with an estimated breeding population of 1–2 million pairs [10]. It is a migratory species, overwintering in southern and western Europe, and North Africa [11]. The common blackbird (*Turdus merula*) and fieldfare (*Turdus pilaris*) are also common species in the country, with the estimated breeding populations of 250–350 and 80–120 thousand pairs, respectively [10]. The local populations of these birds are partially migratory. The redwing (*Turdus iliacus*) is a rare breeding species in Lithuania, and the majority of birds recorded in the country are migrants from northern Europe [11].

A limited number of cases involving *Sarcocystis* spp. in thrushes have been documented in scientific literature. Sarcocysts detected in the common blackbird in Europe were initially described as *S. turdi* [12]. However, this species was later considered invalid due to the absence of detailed morphological characteristics necessary for differentiation [13]. Sarcocysts have also been reported in the muscles of other species of the genus *Turdus*. In Russia, they were observed in fieldfares [14]. In a study in Kazakhstan, infections were found in seven of 88 (8.0%) red-throated thrushes (*Turdus ruficollis*) [15]. In 2012, *Sarcocystis turdusi* was described in the leg muscles of the common blackbird based on light microscopy and transmission electron microscopy (TEM) analyses of isolated sarcocysts and DNA sequence analysis using 18S ribosomal (rRNA), 28S rRNA and ITS1 [16]. To date, *S. turdusi* remains the only valid *Sarcocystis* species associated with the genus *Turdus*. More recently, in a study in Spain, three of 15 (20.0%) song thrushes were found to exhibit *Sarcocystis* sp. infection in the skeletal muscle, with the myocardium being affected in one individual [7]; however, the species was not identified. Recently, sarcocysts have been detected histologically in the muscle tissue of a rufous-bellied thrush (*Turdus rufiventris*) in Brazil [17]. Although the authors suggested an association between these sarcocysts and *S. falcatula*, the absence of molecular analysis, precluded accurate species determination. Within the family Turdidae, infections have also been reported in the genus *Sialia*. Three of 19 (15.8%) Eastern bluebirds (*Sialia sialis*) were found to harbour sarcocysts [18], affecting the skeletal muscle in all cases and the connective tissue associated with the caudal surface of the left eye in one individual. Partial 18S rRNA gene sequences were analysed; however, the parasite could not be conclusively assigned to a single species.

Genetic analyses have demonstrated that birds are both the intermediate and definitive hosts of *S. turdusi*. Molecular studies have detected *S. turdusi* DNA in mucosal scrapings from several avian species belonging to the families Accipitridae and Corvidae [4,19,20,21,22]. However, the detection of parasite DNA alone does not confirm definitive-host status. Thus, the role of corvids as definitive hosts therefore remains uncertain, as only oocysts, but not sporocysts, have been reported. The potential involvement of additional intermediate hosts of *S. turdusi* also remains unclear. To date, data on *Sarcocystis* spp. infections in thrush species other than the common blackbird are limited. Thus, we collected and analysed muscle tissue samples from deceased thrushes of the genus *Turdus* across different regions of Lithuania and Rybachy (Kaliningrad Region, Russia) between 2003 and 2024. The aims of this study were to determine and compare parasite infection prevalence and load, identify *Sarcocystis* species, assess host specificity, and expand the available molecular data on *Sarcocystis* spp. in different thrush species.

## 2. Materials and Methods

### 2.1. Materials

**Figure 1:**
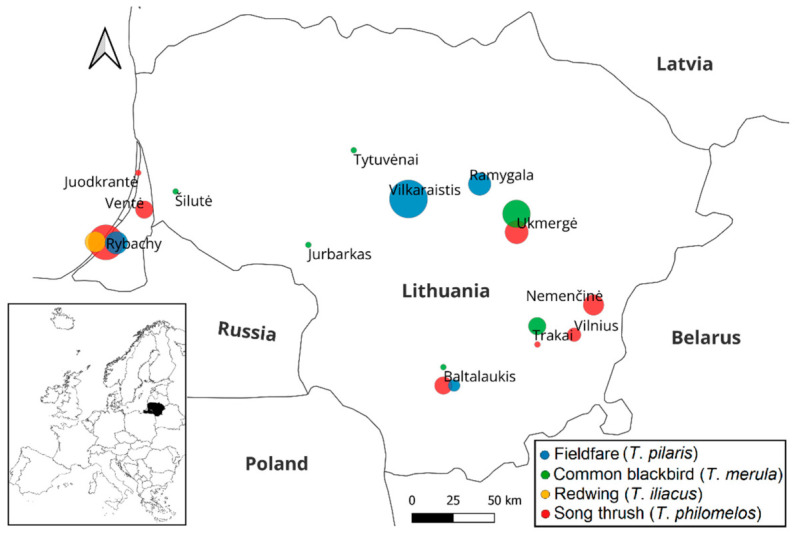
Map showing the locations where samples were collected. Circle size corresponds to the number of birds sampled. The map was created using QGIS (version 3.40.11). Administrative boundary data: World Administrative Boundaries dataset, licenced under the Open Government Licence v3.0.

From 2003 to 2024, a total of 72 samples of breeding and migratory birds of the genus *Turdus* were investigated for the presence of *Sarcocystis* cysts. The samples were collected over time from the following locations: Rybachy (Kaliningrad region, Russia), Juodkrantė, Šilutė and Ventė (Klaipėda County, Lithuania), Ramygala (Panevėžys County, Lithuania), Tytuvėnai (Šiauliai County, Lithuania), Jurbarkas (Tauragė County, Lithuania), Baltalaukis, Nemenčinė, Trakai, Ukmergė, Vilkaraistis and Vilnius (Vilnius County, Lithuania) (Figure 1). All birds were found dead. In Lithuania, the birds were obtained from the Kaunas Tadas Ivanauskas Zoology Museum, the Lithuanian national authority responsible for monitoring dead birds. Samples from the Kaliningrad region of Russia were obtained from the Rybachy Ornithological Station. Birds were delivered to the Laboratory of Molecular Ecology, State Scientific Research Institute Nature Research Centre, Vilnius, Lithuania, for detailed morphological and molecular analysis. The samples were transported in biohazard and transport bags under established temperature protocols at −20 °C to maintain sample integrity and safety. Tissue samples were stored at −20 °C after collection until further analysis. A single freeze–thaw cycle occurred between the initial infection evaluation and subsequent morphological analysis. This study was approved by the Animal Welfare Committee of the SSRI Nature Research Centre (no. GGT-9, issued on 12 January 2024). All procedures were conducted in accordance with applicable laws and ethical standards, and bird remains were properly disposed of following environmental and veterinary regulations.

### 2.2. Study Workflow in Different Periods

**Figure 2:**
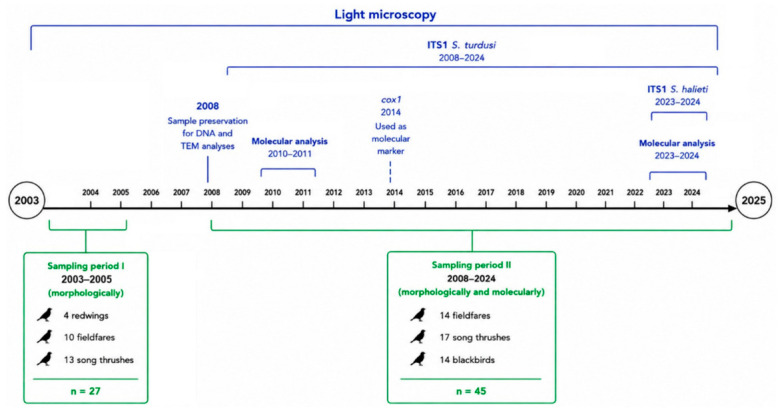
Development of sampling and DNA analysis in thrush species over time.

The timeline illustrates the progression and continuity of research conducted between 2003 and 2024, highlighting key methodological developments and sampling events (Figure 2). Preliminary research (2003–2005) primarily involved morphological parasite analyses across multiple *Turdus* species. From 2008, the study incorporated the preservation of *Sarcocystis* samples for subsequent DNA and TEM analyses. From 2010 onwards, molecular approaches became increasingly prominent, with particularly intensive analyses conducted from 2010 to 2011. The timeline shows the periodic sampling events, indicating the ongoing collection and preservation of deceased birds, predominantly from three thrush species: the common blackbird, song thrush, and fieldfare. Subsequent study phases (2015–2024) incorporated the consistent utilisation of DNA-based techniques, reflecting a transition towards molecular characterisation. We used the cytochrome c oxidase subunit I (*cox1*) gene as a molecular marker (around 2014); however, this gene was later found (2018) to have limited utility for differentiating *Sarcocystis* species in birds as intermediate and definitive hosts. The most recent years (2023–2024) are characterised by another period of intensive genetic analyses.

### 2.3. Microscopic Analysis

In all cases, a thorough examination of the leg muscles was conducted on the avian samples. The prevalence and parasite load of *Sarcocystis* infection were evaluated by light microscopy of methylene blue-stained, squeezed muscle samples. The examination involved counting cysts within approximately 1 g of muscle tissue. In this procedure, the muscle was cut into about 28 pieces, each comparable in size to rice grains. The cut pieces were then placed on a synthetic mesh and immersed for 20–30 min in a 0.2% aqueous methylene blue solution in a Petri dish. Following staining, the muscle pieces, along with the mesh, were placed on filter paper for a few seconds to remove excess dye. The samples were then immersed for 15–20 min in a 1.5% acetic acid solution and gently agitated to separate tissue pieces. The muscle pieces were again placed on filter paper to dry momentarily, then transferred to a compression glass (compressorium), a device comprising two glass plates used to flatten tissue samples for microscopic examination. Samples were examined under a light microscope at ×40 or ×100 magnification. The total number of sarcocysts observed within the compressor fields was recorded. The parasite load was calculated only for infected individuals. A detailed examination of the morphology of sarcocysts was conducted using fresh preparations. The sarcocysts were isolated from the muscle fibres with a pair of preparation needles. A subset of sarcocysts collected from different thrush species was selected for molecular analysis. One representative sarcocyst per bird was selected for molecular analysis based on intact morphology and suitability for DNA extraction.

In 2008, for TEM, a mature sarcocyst containing a small quantity of muscle fibres from a fieldfare was fixed in Karnovsky’s fixative, postfixed in 1% osmium tetroxide, dehydrated and embedded in Epon. Subsequently, ultrathin sections were stained with 2% uranyl acetate and lead citrate and examined by JEOL JEM-100B TEM (JEOL Ltd., Tokyo, Japan). This experiment was conducted commercially at the former Department of Experimental and Clinical Medicine of the Innovative Medicine Centre, Vilnius, Lithuania.

### 2.4. Molecular Analysis

Molecular studies were conducted on two separate occasions over a period of two decades. During the 2010–2011 period, genomic DNA was extracted from seven selected individual sarcocysts using the QIAamp^®^ DNA micro kit (Qiagen, Hilden, Germany), in accordance with the manufacturer’s protocol. Successful DNA extraction was achieved from sarcocysts obtained from two song thrushes, two fieldfares, and three common blackbirds. However, no DNA could be extracted from the infected redwing specimen. Isolates were then subjected to PCR amplification for partial ITS1 and/or *cox1* sequences using P-ITSF/P-ITSR (forward: ATTGAGTGTTCCGGTGAATTA; reverse: GCCATTTGCGTTCAGAAATC) and SF1/SR5 primer pairs, respectively [23]. PCR was performed in a final volume of 25 µL, comprising 1× PCR buffer (with 50 mM KCl), 0.2 mM dNTPs, 0.2 μM of each primer, 2.5 mM MgCl_2_, 1 U Taq DNA polymerase (Thermo Fisher Scientific Baltics, Vilnius, Lithuania), and 0.04 μg of template DNA. The cycling conditions were as follows: initial denaturation at 95 °C for 3 min; followed by 35 cycles of denaturation at 95 °C for 30 s, annealing at 55–60 °C, depending on the primer pair, for 30 s, and extension at 72 °C for 80 s; with a final extension at 72 °C for 7 min.

The second period of molecular analysis was conducted from 2024 to 2025. Genomic DNA was extracted using the GeneJET Genomic DNA Purification Kit (Thermo Fisher Scientific Baltics, Vilnius, Lithuania) according to the manufacturer’s tissue protocol. DNA was successfully extracted from sarcocysts found in four song thrushes and five blackbirds. All isolates were subjected to PCR amplification of the complete or partial ITS1 regions in case the amplification of complete ITS1 was unsuccessful. The complete ITS1 region was amplified using the SU1F/5.8SR2 primer pair [24], and the partial sequences were amplified using the *S. turdusi* species-specific GsSturF/GsSturR primer pair [20]. The PCR reactions were conducted with 2 × Taq Master Mix (Vazyme, Red Maple Hi-tech Industry Park, Nanjing, China) according to the manufacturer’s instructions and specified cycling conditions. The cycling conditions were as follows: initial denaturation at 95 °C for 3 min; followed by 35 cycles of denaturation at 95 °C for 15 s, annealing at 57 °C or 58 °C, depending on the primer pair, for 15 s, and extension at 72 °C for 60 s; with a final extension at 72 °C for 5 min. PCR products were evaluated by 1% agarose gel electrophoresis. Amplified products were purified using ExoI and FastAP (Thermo Fisher Scientific Baltics, Vilnius, Lithuania) and sequenced bidirectionally using a 3500 Genetic Analyzer (Applied Biosystems, Foster City, CA, USA). All sequences generated in the present study are available in GenBank with the following accession numbers KT588510, KJ540164–KJ540166, KT588511–KT588517, PZ364911–PZ364920.

### 2.5. Sequence Analysis

The sequences obtained were compared with those of the *Sarcocystis* species using the online NCBI BLASTn (Nucleotide BLAST) programme (http://blast.ncbi.nlm.nih.gov/, accessed on 2 May 2026). Phylogenetic analyses were performed using MEGA 12.0.14 software [25] based on the ITS1 locus. Phylogenetic trees were constructed using the Maximum Likelihood (ML) method. Only sequences of the same species that differed from one another were selected from the GenBank for phylogenetic analysis. Multiple sequence alignment was generated using the MUSCLE algorithm. The HKY + I nucleotide substitution model was selected as the best model for the dataset analysed based on the lowest Bayesian Information Criterion (BIC) value, calculated using the “Find Best DNA/Protein Models (ML)” function. The robustness of the resulting phylogenetic trees was evaluated using bootstrap analysis with 1000 replicates.

### 2.6. Data Analysis

Statistical analyses of the prevalence and parasite load of *Sarcocystis* spp. were conducted using Quantitative Parasitology 3.0 [26]. Differences in infection prevalence among the four examined thrush species (common blackbird, fieldfare, redwing, and song thrush) were assessed using Fisher’s exact test, and differences in parasite load among these species were evaluated by comparing the median number of sarcocysts using Mood’s median test. A *p* value below 0.05 was considered to indicate significance.

## 3. Results

### 3.1. Prevalence and Parasite Load of Sarcocystis Infection

A total of four host thrush species were examined, and sarcocysts were found in the song thrush, fieldfare, common blackbird, and redwing (Table 1). Among the species studied, the highest prevalence (46.7%) was detected in the song thrush, followed by the common blackbird (42.9%) and fieldfare (29.2%). The redwing had the lowest prevalence (25.0%), with only one infected individual among four examined. The overall prevalence of the infection across all species was moderate, with 38.9% of examined birds affected (28 out of 72 individuals). No significant differences in prevalence were observed among the examined bird species (*p* = 0.600). The number of sarcocysts per one gram of methylene–blue stained muscle samples ranged from 1 to 96. Among the three thrush species with the largest sample sizes, the fieldfare had the highest parasite load (mean = 34). However, no significant differences in parasite load were detected among the species analysed (*p* = 0.207) as median values were similar across species: 8 in the fieldfare, 6.5 in the song thrush, and 5 in the common blackbird.

### 3.2. Morphological Characteristics of Sarcocysts

In total, 23 out of 28 samples were successfully analysed using native preparations across all bird species; however, DNA was not extracted from redwing samples. The thrushes were studied over a long period, and specimens collected in earlier years were analysed only morphologically. However, molecular analysis was introduced in ~2010. Across all four bird species, sarcocysts from 23 birds were analysed morphologically, and molecular analysis was performed on sarcocysts from 13 (Table 2).

**Figure 3:**
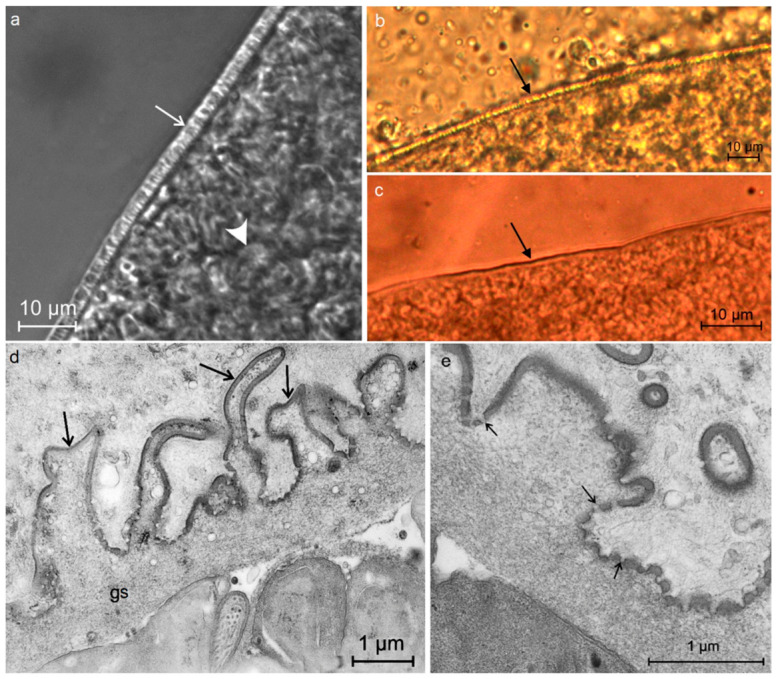
Morphological features of *Sarcocystis* spp. from the leg muscles of thrushes. (**a**–**c**) Light micrographs. Fresh preparations. (**a**) A *S. turdusi* sarcocyst fragment from a fieldfare; the arrow indicates the finger-like protrusions of the sarcocyst wall; note the clearly visible septa (arrowhead). (**b**) A *S. turdusi* sarcocyst fragment from a song thrush; the arrow indicates the finger-like protrusions of the sarcocyst wall. (**c**) A *S. halieti* sarcocyst fragment from a blackbird; the arrow indicates the smooth sarcocyst wall. (**d**,**e**) TEM micrographs of *S. turdusi* sarcocyst wall fragments from a fieldfare. (**d**) Sarcocyst wall protrusions of irregular shapes (arrows). (**e**) High magnification of the base of a villar protrusion. The arrows indicate blebs of parasitophorous vacuolar membrane. Ground substance (gs).

Two morphological types of sarcocysts were found by light microscopy. Predominantly, the sarcocysts observed in the investigated birds corresponded to the morphology described for *S. turdusi*. The sarcocysts were ribbon-shaped, varying in size from 1 to 6 mm in length and 50–280 μm in width. The cyst wall was approximately 3 μm thick and exhibited finger-like protrusions (Figure 3a,b). The internal structure of the sarcocysts was divided into chambers by septa and filled with banana-shaped bradyzoites measuring 6.2 × 1.4 μm (range 5.5–7.1 × 1.2–1.5 μm; n = 20). A second type of microcyst was observed in a single sample (No. NTm13) from an adult male blackbird collected in June 2024 in the Aukštumala raised bog wetland. A small sarcocyst measuring 360 × 40 μm with a smooth cyst wall was observed (Figure 3c). This sarcocyst was assigned to *S. halieti* based on the sequence obtained from the subsequent molecular analysis.

By TEM, the cyst wall of *S. turdusi* from the fieldfare was found to be 2.8–3.9 μm in thickness (including the ground substance) and exhibited villar protrusions that were club- or irregularly shaped and sometimes branched (Figure 3d). The height of the villar protrusions varied between 1.8 and 2.8 μm. At the base, the width of the protrusions ranged from 0.5 to 1.3 μm, and the distances between them were 0.3–0.8 μm. The tops of the protrusions were rounded. The parasitophorous vacuolar membrane had many minute blebs, and the electron-dense layer was interrupted in some areas (Figure 3e). Neither fibrillar elements nor vesicles were observed in the protrusions. The sarcocyst wall was consistent with type 18a of the Dubey et al. [1] classification. TEM analysis was not conducted on sarcocysts with finger-like protrusions from the song thrush, as these were considered to belong to the same *Sarcocystis* species that had been previously characterised. Likewise, as the ultrastructure of *S. halieti* has been described previously [27], it was not examined in this study.

### 3.3. Molecular Analysis of Isolated Sarcocysts

The ITS1 and *cox1* sequences obtained from each host are provided in Table 3. Overall, eight complete ITS1 sequences of *S. turdusi* were obtained from song thrush, fieldfare and common blackbird. Additionally, five partial ITS1 of *S. turdusi* were established. Based on the comparison of complete ITS1 sequences, *S. halieti* was identified in one common blackbird, in which *S. turdusi* was also identified. Finally, seven partial *cox1* sequences of *S. turdusi* were established from the same three thrush species.

**Figure 4:**
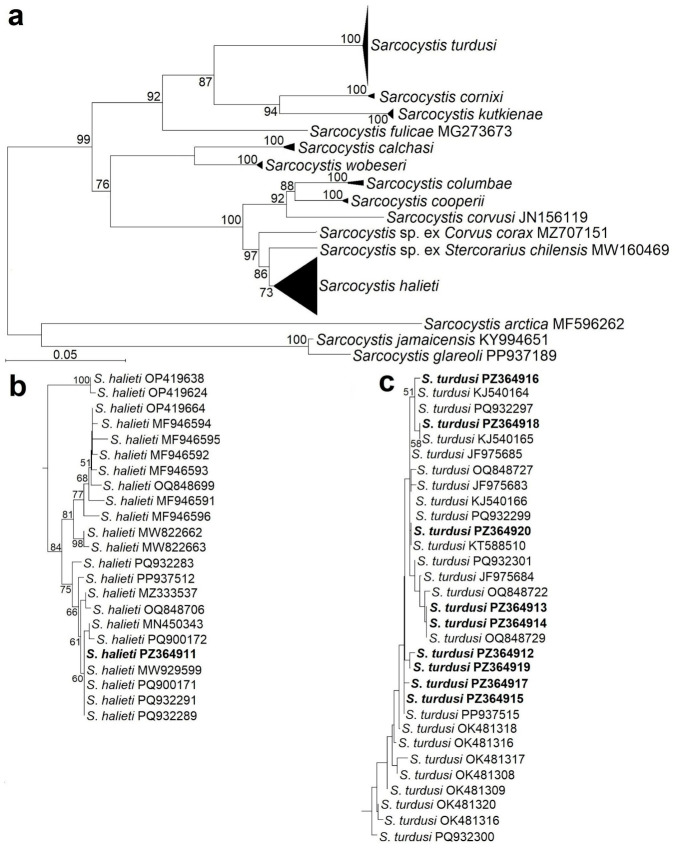
Phylogenetic relationships of two *Sarcocystis* species, *S. halieti* and *S. turdusi* detected in the muscles of thrushes based on ITS1 sequences. (**a**) A complete view of the phylogram, which was constructed using 81 taxa and 670 nucleotide positions. A detailed representation of the phylogenetic relationships between isolates of *S. halieti* (**b**) and *S. turdusi* (**c**). Phylogenetic trees were generated using the ML method, scaled to branch lengths and rooted at *S. arctica*, *S. glareoli* and *S. jamaicensis*. The sequences obtained in the present study are shown in bold.

The 822 bp ITS1 sequence of *S. halieti* from the common blackbird shared 97.3–100% similarity with other conspecific sequences and exhibited very high similarity to *Sarcocystis* sp. ex *Stercorarius chilensis* and *Sarcocystis* sp. ex *Corvus corax* (96.1 and 94.7%, respectively) (Table 4). In the phylogram, our *S. halieti* sequence significantly (with 73 bootstrap value) grouped with other isolates of the same species (Figure 4a,b), supporting the correct identification of *S. halieti*. At ITS1, *S. halieti* was placed together with the two mentioned *Sarcocystis* sp. taxa, as well as with *S. columbae*, *S. cooperii* and *S. corvusi*. Specifically, *Sarcocystis* sp. ex *Stercorarius chilensis* formed a sister clade to *S. halieti*, while *Sarcocystis* sp. ex *Corvus corax* was a sister taxon to this clade.

Our complete (793–794 bp) and partial (511 bp) ITS1 sequences of *S. turdusi* differed from one another by up to 0.9%, showed ≥97.9% similarity to other sequences of the same species and ≤86.1% similarity to sequences of other species available in GenBank (Table 4). Phylogenetic analysis revealed that *S. turdusi* was most closely related to *S. cornixi* and *S. kutkienae*, and no phylogenetic grouping was observed among *S. turdusi* sequences (Figure 4a,c). Thus, both detected species can be reliably identified using ITS1, whereas minor genetic variation (0.1–0.2%) was observed when comparing 1053 bp *cox1* sequences of *S. turdusi* with those of the most closely related *Sarcocystis* spp. associated with birds (Table 4).

## 4. Discussion

### 4.1. Prevalence and Parasite Load of Sarcocystis spp. in Thrushes

In this study, 72 thrushes were examined, and sarcocysts were detected in 28 individuals (38.9%). The prevalence of infection among different bird species ranged from 25.0% to 46.7%. The lowest prevalence was found in the redwing; however, only four migratory individuals were examined. The prevalence observed in this study was slightly lower than that previously recorded in the common blackbird from the same region (54.5%, 24/44) [16]. However, it was higher than that reported in red-throated thrushes (8.0%, 7/88) from Kazakhstan [15], song thrushes (20.0%, 3/15) from Spain [7], and Eastern bluebirds (15.8%, 3/19) from the USA [18]. Similarly, other studies conducted in Lithuania have reported a low to moderate prevalence of infection across various bird species. Sarcocysts were detected in 8.9% (8/90) of raptors, 10.5% (2/19) of great cormorants, 16.6% (25/151) of gulls, 26% (7/27) of Eurasian coots (*Fulica atra*), and 41.5% (44/106) of corvids [28]. Thus, while a moderate prevalence of *Sarcocystis* infection was found in thrushes, the parasite load in the thrushes examined in this study was generally low, which may indicate a relatively balanced host–parasite relationship. Such a relationship could be consistent with long-term coevolution, in which the parasite can persist within the host population without causing evident pathological effects, thereby ensuring its own transmission [29].

### 4.2. Genetic Identification of Sarcocystis spp. in Thrushes

To date, *S. turdusi* has been known only to form sarcocysts in the muscles of the common blackbird. During this study, *S. turdusi* was confirmed in two additional thrush hosts: the fieldfare and the song thrush. Furthermore, sarcocysts morphologically similar to those of *S. turdusi* were also detected in the redwing, although molecular confirmation is required. The literature indicates that *Sarcocystis* spp. infection is common in the Turdidae family; however, our morphological and molecular data suggest an unusual dominance of a single *Sarcocystis* species, *S. turdusi*, within the analysed samples. A survey of *Sarcocystis* in other avian species in Lithuania revealed the presence of three to five distinct *Sarcocystis* species in birds belonging to the families Anatidae, Laridae, and Corvidae [30]. In contrast, only one *Sarcocystis* species, *S. halieti* was identified in Accipitridae and Strigidae raptors from Lithuania [28]. Thus, the predominance of a single species may represent an atypical pattern within the genus *Sarcocystis* in non-raptorial birds.

In previous studies of red-throated thrushes from Kazakhstan, sarcocysts showed morphological features consistent with *S. turdusi*. However, further molecular studies are required to determine whether other thrush species of the family Turdidae can act as intermediate hosts of *S. turdusi*. Investigations conducted in North and South America may provide additional insights into the diversity and host range of *Sarcocystis* spp. infecting Turdidae [17,18]. The analysis of partial 18S rRNA sequences obtained from a bluebird isolate revealed close similarity to those of *S. falcatula*, *S. neurona*, and *Sarcocystis* sp. ex *Psittacus erithacus*. However, precise species identification requires additional molecular markers. Interestingly, a histological specimen of this isolate is preserved in the U.S. National Parasite Collection, and further genetic characterisation of this material would be valuable. These findings suggest that different biogeographical regions may harbour distinct *Sarcocystis* species infecting Turdidae.

Definitive hosts of *S. turdusi* include raptors and corvids from both Europe and North America. In Spain, these hosts comprise the common buzzard (*Buteo buteo*), Eurasian sparrowhawk (*Accipiter nisus*), and northern goshawk (*Accipiter gentilis*) [21]. In Lithuania, hosts include the same raptor species as in Spain, as well as the Eurasian magpie (*Pica pica*), western jackdaw (*Coloeus monedula*), and common raven (*Corvus corax*) [20,22]. In the USA, definitive hosts comprise the northern goshawk, red-tailed hawk (*Buteo jamaicensis*), sharp-shinned hawk (*Accipiter striatus*), and Cooper’s hawk (*Astur cooperii*) [4]. Because suitable definitive hosts occur across broad geographical regions, *S. turdusi* may circulate widely among migratory and resident Turdidae populations in Europe and North America.

The genetic identification of *S. turdusi* and *S. halieti* in the current study was performed using the ITS1 region, amplified with either universal or species-specific primers. This approach was necessary because amplification of the full ITS1 region can be hindered by tandem repeat sequences. However, ITS1 has limited resolution for distinguishing more distantly related taxa. Moderate to high intraspecific genetic variability has been observed in both *S. halieti* and *S. turdusi* in a broader comparative context [30]. Intraspecific variability in *S. turdusi* has been reported to reach up to 1.1%, whereas *S. halieti* exhibits substantially higher variation, with differences of up to 2.9% among isolates. The elevated intraspecific genetic variability observed in *S. halieti* is likely associated with its broad geographic distribution and wide range of intermediate hosts. In this study, intraspecific variability within *S. turdusi* ITS1 sequences reached 1.1% when examining complete sequences. The variability was even higher when only partial ITS1 sequences were analysed, reaching 2.1%. Thus, the relatively high genetic variability observed in *S. turdusi* may suggest that this species is not restricted to a single host genus but could occur across multiple members of the Turdidae family or even other passerine birds. This hypothesis is further supported by the currently available GenBank record, which indicates the presence of *S. turdusi* in an additional host species, the European robin (*Erithacus rubecula*) of the family Muscicapidae (accession no. KJ540167). Nevertheless, further investigations involving a broad range of Passeriformes species are needed to disclose the host spectrum and genetic diversity of *S. turdusi*.

### 4.3. The Significance of the Detection of S. halieti in Thrushes

*Sarcocystis halieti* is notable for its broad range of intermediate hosts. The species was first described relatively recently, in 2018 [31], and has since been detected in the muscles of birds belonging to the orders Accipitriformes, Passeriformes, Charadriiformes, Suliformes, Procellariiformes, and Strigiformes [3,27,30,32,33]. The occurrence of this species has also been documented across several continents, including Europe (Czech Republic, Germany, Lithuania, Norway, Spain, and Greece), South America (Brazil), North America (USA), Asia (Iran), and Africa (Egypt).

In the present study, *S. halieti* was detected in an atypical host species. However, this appears to represent an isolated case, as the species was recorded in only one individual during the long-term survey. This may be related to the individual condition of the host. Both *S. turdusi* and *S. halieti* were identified in this bird. It is known that both species utilise Accipitridae as definitive hosts. Thus, this finding likely represents a sporadic occurrence. However, further studies with larger sample sizes are required to assess its epidemiological relevance. The definitive hosts of *S. halieti* have been identified in raptors in Lithuania, and the parasite has also been reported in raptors from Spain and the USA [4,20,21,22]. Surveillance of definitive hosts should be included in future monitoring programmes to assess potential geographic and host range expansion of *S. halieti*. Such studies would improve our understanding of ecology, transmission dynamics, and the possible spread of this parasite into new regions. At present, only a limited number of avian-pathogenic *Sarcocystis* species, including *S. neurona* and *S. calchasi*, have been characterised at the genomic and molecular levels [34,35]. Available whole-genome or draft-genome data for these species provide insight into their potential neurotropic properties and pathogenicity. Given the currently limited genomic resources, there is a need for expanded genomic and transcriptomic data to improve the resolution of avian-associated *Sarcocystis* species. As *S. halieti* may be a potentially pathogenic species, further investigation of its genetic diversity, life cycle, and host associations is essential to evaluate its veterinary significance.

## 5. Conclusions

This study expands current knowledge of *Sarcocystis* infections in thrushes, indicating a moderate prevalence of sarcocysts in sampled dead birds and identifying *S. turdusi* as the dominant species across multiple host species. The detection of this parasite in the song thrush and fieldfare suggests that these birds may act as new intermediate hosts, contributing to a more comprehensive understanding of host–parasite associations within this genus. Molecular analyses demonstrated that ITS1 is a suitable marker for species-level identification, whereas *cox1* showed limited discriminatory power for *Sarcocystis* spp. in birds, which serve as both intermediate and definitive hosts. The detection of *S. halieti* in a common blackbird represents a novel and atypical host record; however, its epidemiological significance remains uncertain and requires confirmation through further studies. The observed patterns, including species prevalence and host associations, should be interpreted with caution due to the opportunistic sampling of birds found dead, unequal sample sizes, and limited molecular confirmation. Overall, these findings highlight the complexity of *Sarcocystis* transmission dynamics in wild birds and underline the importance of integrative approaches for assessing parasite diversity and host specificity.

## Figures and Tables

**Table 1:** Infection parameters of *Sarcocystis* in thrush (*Turdus* spp.) methylene blue-stained muscle samples.

Bird Species	Number of Birds Infected/Investigated (%)	Parasite Load (Number of Sarcocysts)	Mean Parasite Load	Median Parasite Load
Song thrush (*T. philomelos*)	14/30 (46.7%)	1–46	12.9	6.5
Fieldfare (*T. pilaris*)	7/24 (29.2%)	1–96	34.0	8.0
Common blackbird (*T. merula*)	6/14 (42.9%)	1–14	5.8	5.0
Redwing (*T. iliacus*)	1/4 (25%)	66	66.0	66.0
Total	28/72 (38.9%)	1–96	18.5	6.5

**Table 2:** Overview of the sample size, sarcocyst detection in microscopic preparations, and the number of sarcocysts isolated for *Sarcocystis* species identification.

Bird Species	Sample Size (n)	Sarcocysts in Fresh/ Stained Preparations	Number of Sarcocysts Isolated for DNA Analysis *
Song thrush (*T. philomelos*)	30	10/14	6
Fieldfare (*T. pilaris*)	24	6/7	2
Common blackbird (*T. merula*)	14	6/6	5
Redwing (*T. iliacus*)	4	1/1	0

* In cases when molecular analysis was performed, a single sarcocyst was isolated from each bird.

**Table 3:** Thrush species, the number of birds analysed, methodological approaches, and the GenBank accession numbers of the ITS1 and *cox1* sequences of *Sarcocystis* spp.

Bird Species	Complete ITS1	Partial ITS1	Partial *cox1*
Song thrush (*T. philomelos*)	KJ540166, KT588510, PZ364914, PZ364915	PZ364919, PZ364920	KT588516, KT588517
Fieldfare (*T. pilaris*)	KJ540164, KJ540165		KT588514, KT588515
Common blackbird (*T. merula*)	**PZ364911**, PZ364912, PZ364913	PZ364916–PZ364918	KT588511–KT588513 *

* Additional DNA sequences from a previous study [16]. Notably, the PZ364911 and PZ364912 sequences were obtained from the same bird. The sequence in bold represents *S. halieti*; the other sequences were assigned to *S. turdusi*.

**Table 4:** Molecular characterisation of *S. turdusi* and *S. halieti* isolated from the muscles of thrushes based on ITS1 and *cox1* sequences.

Species	Locus	Similarity Among Isolates (%)	Intraspecific Similarity (%) *	Interspecific Similarity Compared to Closely Related Species (%)
*S. halieti*	ITS1 complete	–	97.3–100	*Sarcocystis* sp. ex *Stercorarius chilensis* (MW160469) 96.1, *Sarcocystis* sp. ex *Corvus corax* (MZ707151) 94.7, *Sarcocystis cooperii* 93.1–93.3, *S. columbae* 92.2–92.5
*S. turdusi*	ITS1 complete	99.1–100	98.9–99.8	*S. fulicae* 85.5–86.1, *S. cornixi* 85.7–85.9, *S. kutkienae* 84.6–85.3, *S. wobeseri* 84.0–84.9
	ITS1 partial	99.2–99.6	97.9–100	*S. wobeseri* 84.4–85.2, *S. kutkienae* 82.6–85.2, *S. cornixi* 83.2–84.0
	*cox1* partial	100	100	*S. cornixi* 99.9, *S. fulicae* 99.9, *S. columbae* 99.8, *S. halieti* 99.8, *S. corvusi* 99.8

* Comparison with sequences of the same species retrieved from GenBank.

## Data Availability

The sequences of *Sarcocystis* were submitted to the NCBI GenBank database under accession numbers KT588510, KJ540164–KJ540166, KT588511–KT588517, PZ364911–PZ364920.

## References

[1] Dubey J.P., Calero-Bernal R., Rosenthal B., Speer C.A., Fayer R. (2016). Sarcocystosis of Animals and Humans.

[2] Fayer R. (2004). *Sarcocystis* spp. in Human Infections. Clin. Microbiol. Rev..

[3] Maier-Sam K., Kaiponen T., Schmitz A., Schulze C., Bock S., Hlinak A., Olias P. (2021). Encephalitis Associated with *Sarcocystis halieti* Infection in a Free-Ranging Little Owl (*Athene noctua*). J. Wildl. Dis..

[4] Rogers K.H., Arranz-Solís D., Saeij J.P.J., Lewis S., Mete A. (2022). *Sarcocystis calchasi* and Other Sarcocystidae Detected in Predatory Birds in California, USA. Int. J. Parasitol. Parasites Wildl..

[5] Himmel T., Harl J., Pfanner S., Nedorost N., Nowotny N., Weissenböck H. (2020). Haemosporidioses in Wild Eurasian Blackbirds (*Turdus merula*) and Song Thrushes (*T. philomelos*): An in Situ Hybridization Study with Emphasis on Exo-Erythrocytic Parasite Burden. Malar. J..

[6] Llanos-Soto S., Córdoba M., Moreno L., Kinsella J.M., Mironov S., Cicchino A., Barrientos C., Martín-Ordenes J.S., González-Acuña D. (2019). External and Intestinal Parasites of the Austral Thrush *Turdus falcklandii* (Aves, Turdidae) in Central Chile. Rev. Bras. Parasitol. Vet..

[7] Cardells-Peris J., Gonzálvez M., Ortega-Porcel J., de Ybáñez M.R.R., Martínez-Herrero M.C., Garijo-Toledo M.M. (2020). Parasitofauna Survey of Song Thrushes (*Turdus philomelos*) from the Eastern Part of Spain. Parasitol. Int..

[8] Deoniziak K., Cichowska A., Niedźwiecki S., Pol W. (2022). Thrushes (Aves: Passeriformes) as Indicators of Microplastic Pollution in Terrestrial Environments. Sci. Total Environ..

[9] Souto H.N., De Campos Júnior E.O., Siqueira M.V.B.M., Campos C.F., Morais C.R., Pereira B.B., Morelli S. (2025). Birds as Environmental Bioindicators of Genotoxicity in Brazilian Cerrado Farmlands: An In Situ Approach. Animals.

[10] Kurlavičius P., Preikša Ž., Skuja S. (2006). Lithuanian Breeding Bird Atlas.

[11] Logminas V. (1990). Birds.

[12] Levine N.D. (1986). The Taxonomy of *Sarcocystis* (Protozoa, Apicomplexa) Species. J. Parasitol..

[13] Odening K. (1998). The Present State of Species-Systematics in *Sarcocystis* Lankester, 1882 (Protista, Sporozoa, Coccidia). Syst. Parasitol..

[14] Grikienienė J., Iezhova T. The prevalence of *Sarcocystis* in some wild birds and poultry in Lithuania and in neighbouring territories. Proceedings of the Special Symposium on Ecology of Bird–Parasite Interactions.

[15] Pak S.M., Eshtokina N.V. (1984). Sarcosporidians of birds. Sarcosporidians of Animals in Kazakhstan.

[16] Kutkienė L., Prakas P., Butkauskas D., Sruoga A. (2012). Description of *Sarcocystis turdusi* sp. nov. from the Common Blackbird (*Turdus merula*). Parasitology.

[17] Ortúzar-Ferreira C.N., Gredilha-Duarte R., Cid G.D.C., Berto B.P., Lopes C.W.G. (2026). Developmental Stages of *Sarcocystis* spp. in Wild Birds from Southeastern Brazil, with a Review of Accipitriformes-Associated Species. Braz. J. Vet. Med..

[18] Carleton R.E., Mertins J.W., Yabsley M.J. (2012). Parasites and Pathogens of Eastern Bluebirds (*Sialia sialis*): A Field Survey of a Population Nesting Within a Grass-Dominated Agricultural Habitat in Georgia, U.S.A., with a Review of Previous Records. Comp. Parasitol..

[19] Mayr S.L., Maier K., Müller J., Enderlein D., Gruber A.D., Lierz M. (2016). Accipiter Hawks (Accipitridae) Confirmed as Definitive Hosts of *Sarcocystis turdusi*, *Sarcocystis cornixi* and *Sarcocystis* sp. Ex *Phalacrocorax carbo*. Parasitol. Res..

[20] Juozaitytė-Ngugu E., Švažas S., Šneideris D., Rudaitytė-Lukošienė E., Butkauskas D., Prakas P. (2021). The Role of Birds of the Family Corvidae in Transmitting *Sarcocystis* Protozoan Parasites. Animals.

[21] Juozaitytė-Ngugu E., Švažas S., Bea A., Šneideris D., Villanúa D., Butkauskas D., Prakas P. (2025). Molecular Confirmation of Raptors from Spain as Definitive Hosts of Numerous *Sarcocystis* Species. Animals.

[22] Šukytė T., Butkauskas D., Juozaitytė-Ngugu E., Švažas S., Prakas P. (2023). Molecular Confirmation of Accipiter Birds of Prey as Definitive Hosts of Numerous *Sarcocystis* Species, Including *Sarcocystis* sp., Closely Related to Pathogenic *S. calchasi*. Pathogens.

[23] Gjerde B. (2013). Phylogenetic Relationships among *Sarcocystis* Species in Cervids, Cattle and Sheep Inferred from the Mitochondrial Cytochrome c Oxidase Subunit I Gene. Int. J. Parasitol..

[24] Gjerde B. (2014). Molecular Characterisation of *Sarcocystis rileyi* from a Common Eider (*Somateria mollissima*) in Norway. Parasitol. Res..

[25] Kumar S., Stecher G., Suleski M., Sanderford M., Sharma S., Tamura K. (2024). MEGA12: Molecular Evolutionary Genetic Analysis Version 12 for Adaptive and Green Computing. Mol. Biol. Evol..

[26] Reiczigel J., Marozzi M., Fábián I., Rózsa L. (2019). Biostatistics for Parasitologists—A Primer to Quantitative Parasitology. Trends Parasitol..

[27] Prakas P., Butkauskas D., Švažas S., Stanevičius V. (2018). Morphological and Genetic Characterisation of *Sarcocystis halieti* from the Great Cormorant (*Phalacrocorax carbo*). Parasitol. Res..

[28] Prakas P., Šukytė T., Juozaitytė-Ngugu E., Butkauskas D. (2025). Detection of *Sarcocystis halieti* in Muscles of Raptors from Lithuania. Front. Vet. Sci..

[29] Schmid-Hempel P. (2021). Evolutionary Parasitology: The Integrated Study of Infections, Immunology, Ecology, and Genetics.

[30] Prakas P., Calero-Bernal R., Dubey J.P. (2025). *Sarcocystis* Infection in Domestic and Wild Avian Hosts: Inseparable Flight Partners. Vet. Parasitol..

[31] Gjerde B., Vikøren T., Hamnes I.S. (2018). Molecular Identification of *Sarcocystis halieti* n. sp., *Sarcocystis lari* and *Sarcocystis truncata* in the Intestine of a White-Tailed Sea Eagle (*Haliaeetus albicilla*) in Norway. Int. J. Parasitol. Parasites Wildl..

[32] Máca O., González-Solís D. (2022). Role of Three Bird Species in the Life Cycle of Two *Sarcocystis* spp. (Apicomplexa, Sarcocystidae) in the Czech Republic. Int. J. Parasitol. Parasites Wildl..

[33] Llano H.A.B., Zavatieri Polato H., Borges Keid L., Ferreira De Souza Oliveira T.M., Zwarg T., De Oliveira A.S., Sanches T.C., Joppert A.M., Gondim L.F.P., Martins Soares R. (2022). Molecular Screening for Sarcocystidae in Muscles of Wild Birds from Brazil Suggests a Plethora of Intermediate Hosts for *Sarcocystis falcatula*. Int. J. Parasitol. Parasites Wildl..

[34] Maier-Sam K., Rupp O., Voss A., Nemitz S., Wollenweber T.E., Procida-Kowalski T., Wilhelm J., Gruber A.D., Lierz M. (2025). Differential Expression of Host Invasion-Associated Genes by *Sarcocystis calchasi* in Intermediate versus Definitive Hosts. PLoS ONE.

[35] Dangoudoubiyam S., Norris J.K., Namasivayam S., De Paula Baptista R., Cannes Do Nascimento N., Camp J., Schardl C.L., Kissinger J.C., Howe D.K. (2024). Temporal Gene Expression during Asexual Development of the Apicomplexan *Sarcocystis neurona*. mSphere.

